# Utility of Oblique Sagittal Reformatted and Three-dimensional Surface Reconstruction Computed Tomography in Foraminal Stenosis Decompression

**DOI:** 10.1038/s41598-018-34458-9

**Published:** 2018-10-30

**Authors:** Masahito Oshina, Yasushi Oshima, Sakae Tanaka, Lee A. Tan, Xudong Josh Li, Alexander Tuchman, K. Daniel Riew

**Affiliations:** 1 Department of Orthopaedic Surgery, The University of Tokyo Hospital 7-3-1, Hongo, Bunkyo-Ku, Tokyo 113-8655 Japan; 2 Department of Orthopedic Surgery, Columbia University/The Allen Hospital, 5141 Broadway, New York, New York 10034 United States

## Abstract

Determining the responsible level of cervical radiculopathy can be difficult. Because asymptomatic findings are common in cervical radiculopathy, diagnoses based on imaging studies can be inaccurate. Therefore, we investigated whether the application of oblique sagittal reformatted computed tomography (oblique sagittal CT) and three-dimensional surface reconstruction CT (3DCT) affects surgical plans for patients with cervical foraminal stenosis and whether it assists diagnosis of foraminal stenosis. Accordingly, four reviewers, with office notes, observed the CT and magnetic resonance imaging (MRI) images of 18 patients undergoing surgical treatment for cervical radiculopathy. After reviewing the MRI and sagittal, coronal, and axial CT images, the reviewers recorded the operation to be performed; they examined oblique sagittal CT and 3DCT images of the same patients and noted any differences from their surgical plans. Consequently, we analyzed these changes in the decompressed foramina in the surgical plan; mean percent change in the plan was 18.1%. Inter-rater reliability improved from κ - 0.194 to κ - 0.240. Therefore, the addition of oblique and 3DCT images improves inter-rater reliability owing to changes in a part of decompressed foramina. The addition of oblique sagittal CT and 3DCT is helpful in evaluating the foramen and planning surgical treatment of cervical radiculopathy.

## Introduction

Determining the responsible level of cervical radiculopathy can be difficult because radicular pain does not always follow commonly used dermatomal maps^1–3^. On the other hand, diagnoses based on imaging studies can be inaccurate because asymptomatic findings are common^4–6^. Therefore, the surgeon has to rely on a combination of symptoms, physical findings, and concordant imaging findings to determine the source of the patient’s symptoms. Occasionally, the imaging findings can be subtle and are missed without a proper plane of view. Foramina, in particular, have complicated shapes. As per our experience, determination of foraminal narrowing can often be improved^7^ by several different modalities, including plain radiographs, computed tomography (CT) scans, and magnetic resonance imaging (MRI) in multiple planes.

Although few studies have evaluated the utility of oblique sagittal reformatted CT (oblique sagittal CT) images in assessing cervical neuroforaminal stenosis^8,9^, to the best of our knowledge, no study has reported the extent to which oblique sagittal CT and three-dimensional surface reconstruction CT (3DCT) affect the surgical plan in the cervical spine. The purpose of this study was to determine whether using oblique sagittal CT and 3DCT affects surgical plans for patients with cervical foraminal stenosis.

## Results

In the 18 cases, the addition of the two imaging modalities resulted in a mean absolute percent change in the surgical plan of 18.1% (11%–41%). Fusion was added in cases of anterior cervical decompression and fusion and not added in cases of laminoplasty. Of note, after examining oblique sagittal CT and 3DCT images, every reviewer’s number of decompressed foramina increased. A total of 24 foramina among the 18 patients were additionally decompressed by the four reviewers and degree of coincidence was increased.

According to the Landis and Koch score, inter-rater reliability was “slight” when axial, sagittal, and coronal CT and axial and sagittal MR images were reviewed (κ - 0.194) and improved to “fair” when adding reformatted oblique sagittal CT and 3DCT images (κ - 0.240) (Table 1).

**Table 1:** Kappa–Fleiss coefficient and Landis–Koch Score when all four reviewers were compared for each imaging modality.

	Kappa–Fleiss coefficient	Landis–Koch Score
CT (axial, coronal, and sagittal) and MRI (axial and sagittal)	0.194	slight agreement (0.10–0.20)
CT (axial, coronal, and sagittal), MRI (axial and sagittal), and oblique sagittal CT, 3DCT	0.240	Fair agreement (0.21–0.40)

CT; computed tomography, MRI; magnetic resonance imaging, oblique sagittal CT; oblique sagittal reformatted computed tomography, 3DCT; 3-dimensional surface reconstruction computed tomography.

**Figure 1:**
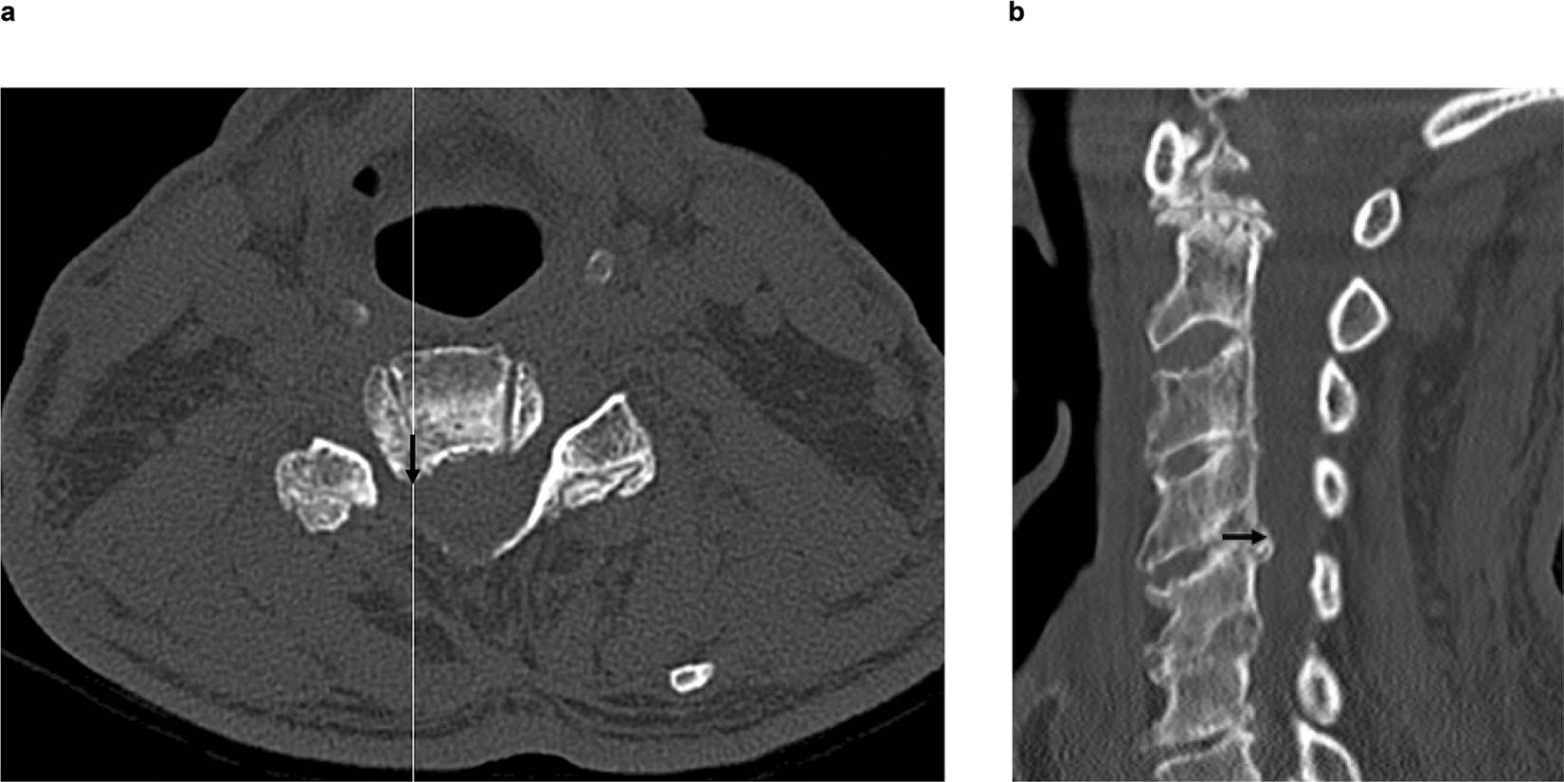
(**a**) Axial CT: Line is a sagittal slice direction; arrow is an osteophyte direction. (**b**) Sagittal CT: Arrow is an osteophyte direction. We can clearly recognize the bony spur in the parasagittal view.

**Figure 2:**
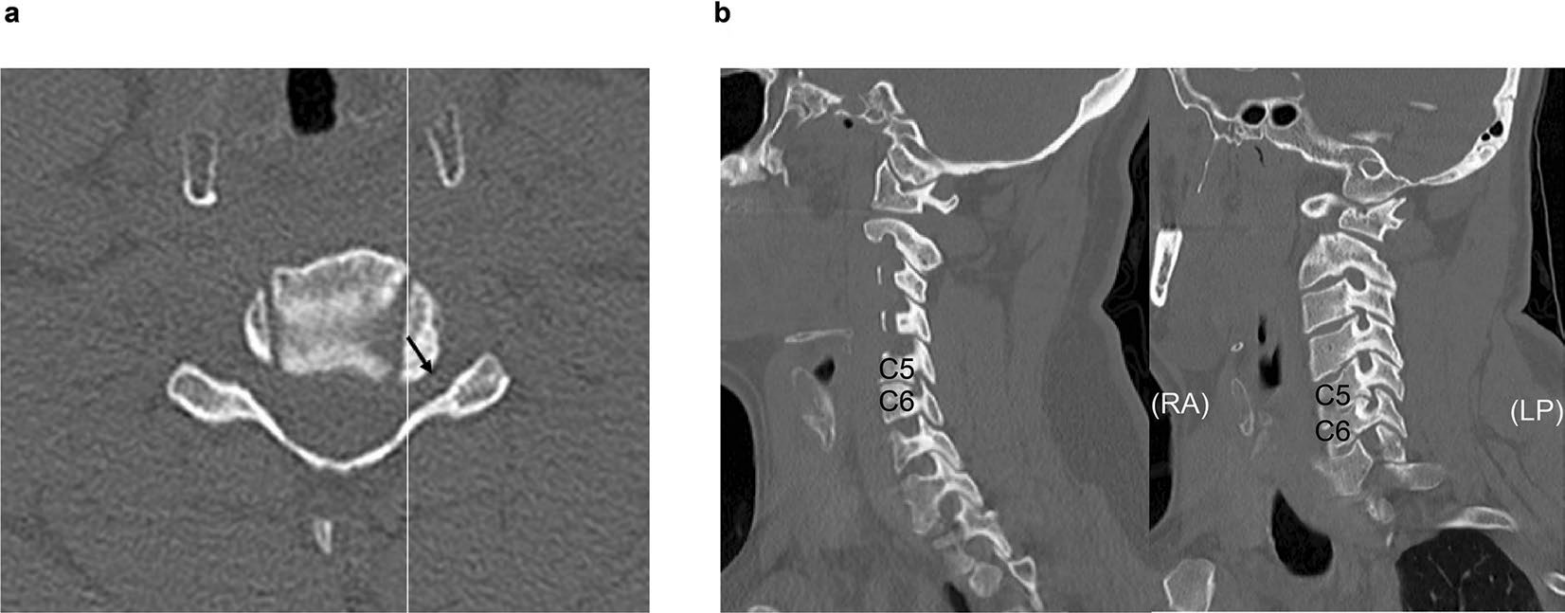
(**a)** Axial CT at C5/6: Line is a sagittal slice direction; arrow is an osteophyte direction. (**b**) Left: Foraminal narrowing at C5/6 in normal sagittal CT; Right: Foraminal narrowing at C5/6 in oblique reformatted sagittal CT. Both were observed in the same patient, but the normal sagittal view showed less foraminal narrowing.

When the type of foraminal stenosis was divided into two areas, at the entrance to the canal and within the foraminal canal^10^, we found that the additional images were particularly useful when the foraminal stenosis was not located at the entrance to the canal (Fig. 1a,b) but located within the foraminal canal (Fig. 2a,b). In fact, of a total of 24 decompressed foramina, 23 were within the foraminal canal and only one foramen was at the entrance to the canal.

## Discussion

MRI is an important modality for evaluating neural compression in the cervical spine. However, it cannot always differentiate soft tissues from bony pathology in the foramen^11^; therefore, an additional evaluation of CT images can be helpful in surgical planning. CT is helpful in identifying osteophytes in the spondylotic spine, whereas MRI is helpful for identifying foraminal stenosis because of the involvement of soft tissue^12–14^. However, the frequency of radiculopathy depends more on osteophytes than on disk herniation^15^. Because evaluating bony foraminal stenosis is important in formulating a surgical plan, we sought to determine the utility of oblique sagittal CT and 3DCT images in identifying bony foraminal stenosis in surgical cases.

We found that the addition of oblique sagittal CT and 3DCT images substantially changed the surgical plans of all four surgeons. After reviewing the additional images, every reviewer decided to decompress the additional foramina, thus resulting in a total of 24 additional foramina being decompressed for the 18 patients. The surgical plans involving the decompressed foramina changed on an average in 18.1% of the cases (11%–41%). We also found that the additional images improved inter-rater reliability by one grade: from “slight” to “fair.” This indicates that even with a full set of MR images; plain radiographs; and axial, sagittal, and coronal CT images, the addition of oblique sagittal CT and 3DCT images can alter surgical treatment plans in patients with foraminal stenosis. In retrospect, regarding the finding that the number of decompression sites increased, although clinical findings were noted, the sites at which no stenosis was detected on primary imaging were those at which decompression was added. Dermatome and myotome maps occasionally overlap at each nerve root^2^; therefore, it is difficult to be certain regarding the responsible nerve root based solely on physical findings. In such cases, the extended protocol facilitates bridging of the gap between physical and imaging findings. Therefore, we believe that the findings observed in the extended protocol agreed better with the pre-surgical clinical symptoms.

**Figure 3:**
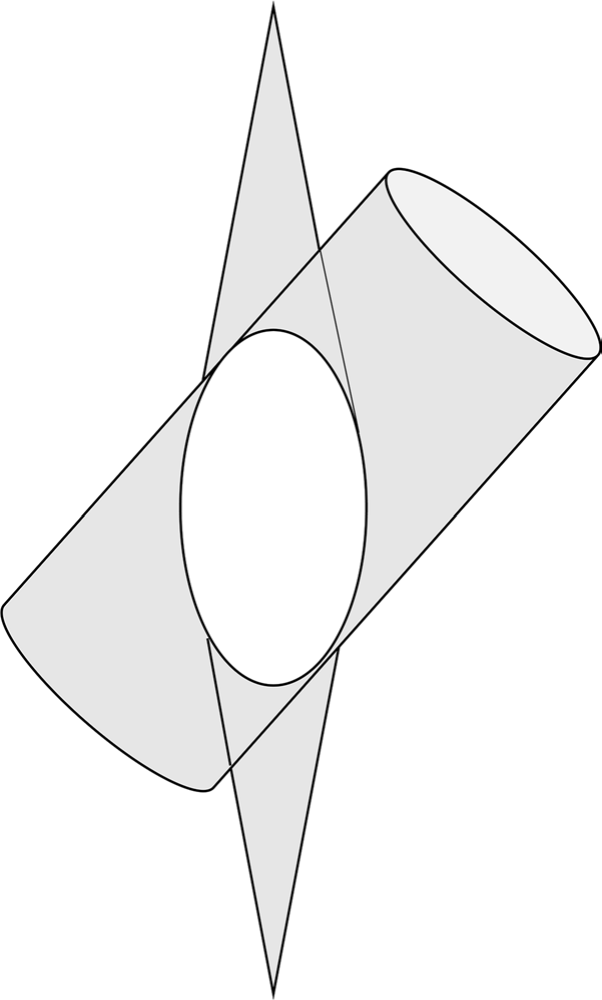
Standard sagittal images cut oblique to the cylinder; therefore, each image is oval.

There are a number of reasons for why foraminal narrowing is better appreciated on oblique sagittal CT and 3DCT images. First, in the stenosis area at the entrance to the canal, the osteophyte projects directly posteriorly in the same plane as the sagittal slice. Therefore, there is minimal advantage to the additional images (Fig. 1). In contrast, foraminal narrowing due to intraforaminal osteophytes was better appreciated on the oblique sagittal images. This is because the foraminal canal is oriented in an oblique angle. Therefore, an oblique sagittal image is required to see the foramen in perfect cross section. If we consider foramen as a cylinder, oblique sagittal images cut perpendicular to the cylinder such that each image is perfectly circular. However, standard sagittal images cut oblique to the cylinder such that each image is oval (Fig. 3). Therefore, the foramen space was observed as enlarged and emphasized in standard sagittal images owing to the fact that an oval is larger than a circle (Fig. 2).

Second, in this study, the CT cutline had a slice thickness of 2.0 mm and a 2.0-mm interval; an axial image just above or below the area of the greatest foraminal compression could easily miss the diagnosis.

Our study is similar to previous work demonstrating that oblique sagittal MRI scans provide useful information^16–19^. Oblique MRIs were useful in demonstrating foraminal disk herniations and foraminal stenosis in the same way, and we found that bony foraminal stenosis was better demonstrated on oblique sagittal CT images than straight sagittal ones. CT can be reconstructed in other planes after obtaining the initial CT imaging data, without adding to the patient’s burden; adding other views by performing oblique sagittal CT and 3DCT can easily increase the available information on the foramen.

There are several limitations to this study. First, other than plain radiographs, neither CT nor MRI is a weight-bearing upright image. Therefore, it is possible that studies on upright images yield different data. Second, we limited the study to cases where MRI and CT were performed within 4 months of each other. It is possible that a new pathology occurred in the interim. However, because we compared the addition of oblique sagittal CT and 3DCT images to standard CT images, which were obtained at the same time, it is difficult to argue that the time difference between CT and MRI had any substantial effect on the results. Third, we did not evaluate the effect of age on the results of the study but believe that the greater the bony foraminal stenosis the greater the benefits of obtaining the additional images. Fourth, we could not manipulate the angle of the oblique sagittal CT or 3DCT images, which some PACSs can now do. This should allow for surgeons to obtain even more useful information than what we found by showing only one oblique angle. Fifth, we combined the use of oblique sagittal CT and 3DCT images rather than evaluating each separately. This is because both are readily available with most PACSs and do not require additional data acquisition. Because our purpose was to evaluate the additional benefits of using all planar images that are available to many surgeons, we felt that it was not needed to study each separately. Finally, although addition of oblique sagittal CT and 3DCT images is valuable in determining the surgical plan, our study did not present intraoperative findings or data on clinical outcomes that would confirm the diagnostic accuracy, and there may be a risk of over surgical treatment when relying too much on the results of imaging studies. It is important that each surgeon assesses the findings of MRI and physical examination and clinical symptoms. Whether this actually improves the clinical outcome has to be further assessed in future studies.

In conclusion, to the best of our knowledge, this is the first study to investigate the utility of oblique sagittal CT and 3DCT in surgical planning for patients with cervical radiculopathy because of foraminal stenosis. We found that oblique sagittal CT and 3DCT increased the surgeons’ consensus on image interpretation of foraminal stenosis, resulting in changes in their surgical plans. In addition, CT and 3DCT enhanced inter-rater reliability compared with only axial, sagittal, and coronal planes. Because adding the oblique sagittal and 3D surface views requires no additional medical cost or radiation to the patient in cases where a CT image has already been obtained, we support the routine use of oblique sagittal CT and 3DCT in surgical planning of patients with cervical radiculopathy.

## Methods

This study was designed as a prospective imaging and surgical procedure analysis and cohort study. We retrospectively analyzed 18 adult patients (11 males, 7 females) who were planning to undergo surgical treatment for cervical spondylotic radiculopathy or myeloradiculopathy from June 1, 2016 to November 30, 2016. The mean age was 61.6 years, with a range of 44–75 years.

Demographic and imaging data were collected from a review of patient charts and a picture archiving and communication system (PACS) at our institution. Basic demographic information such as age, sex, spinal pathology, and cervical spine surgery history were collected from an electronic medical record. CT images were obtained on a 64-slice CT system (60–80 mAs, 120 kVp, FC86 reconstruction kernel, and 2.0-mm slice thickness at 2.0-mm intervals), and MR images were all T2-weighted sequences without T1-weighting obtained on a 1.5-Tesla system. Inclusion criteria were a degenerative condition and planned spinal surgery levels between C2 and T2. Patients were also required to have had preoperative CT and MRI performed within 4 months of each other. Exclusion criteria were age <30 years and having undergone surgery only for soft disk herniation so as to focus on those with degenerative or bony foraminal narrowing.

The data were collected and reviewed by independent researchers after an approval for the protocol was obtained from the institutional review board at our institution. Four reviewers with >5-year experience of spinal surgery were included: one spine surgeon, two neurosurgery fellows and one orthopedic spine fellow; these reviewers assessed the patients’ images and visit notes. They were then asked as to how they would go about performing the surgery.

**Figure 4:**
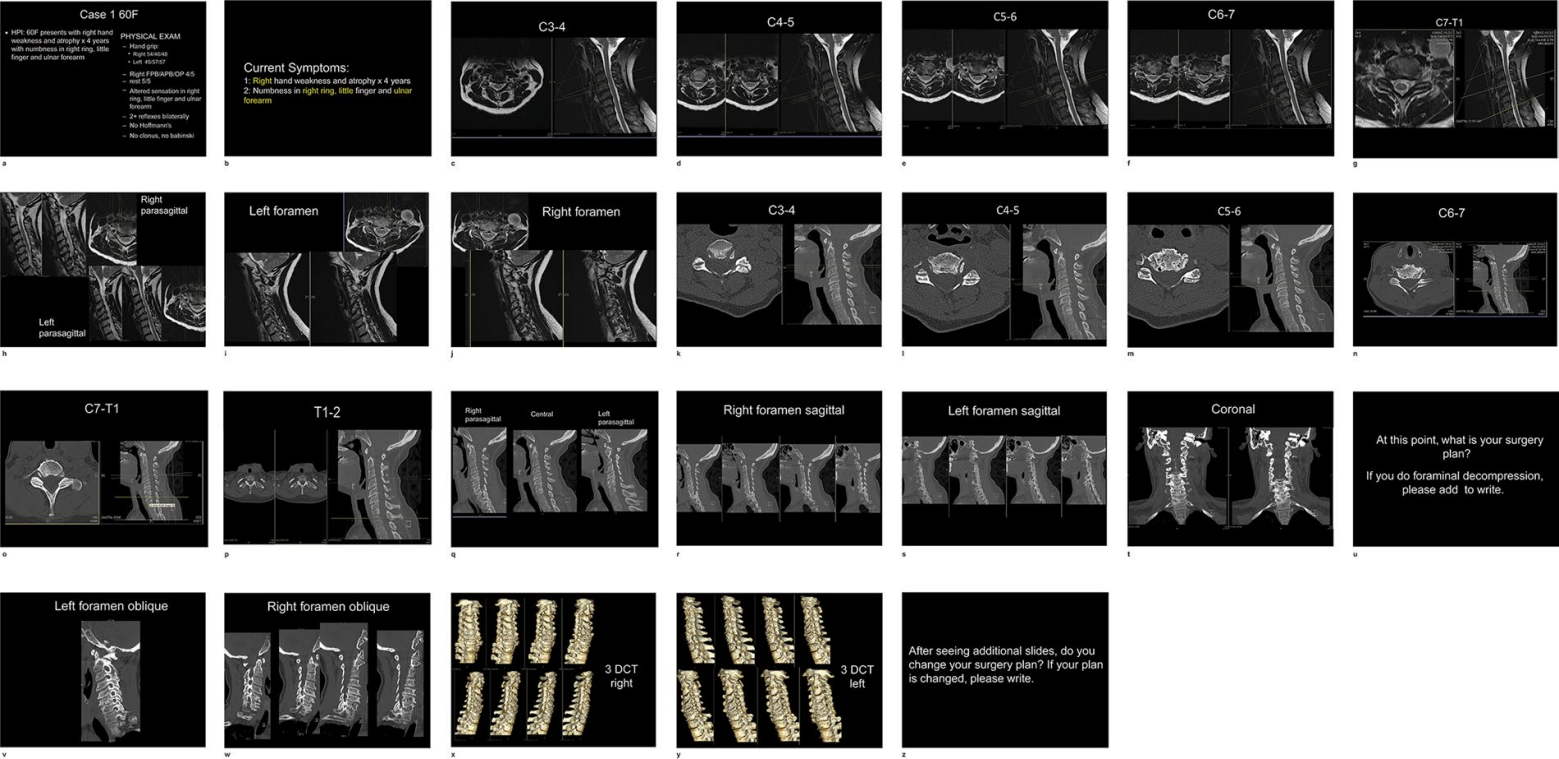
Powerpoint slides presenting the case scheduled for surgical treatment for cervical radiculopathy or myeloradiculopathy.

The study protocol was as follows:

First, the reviewers were shown the sagittal, coronal and axial CT and MRI images including the normal slices of the foramen along with the visit notes. Visit notes included patient’s clinical symptoms and physical examination findings. At this point, the reviewers recorded the type of surgery they would perform and which foramen should be decompressed (e.g. anterior cervical discectomy and fusion C5-C7 with right foraminal decompression C5-7). They were then shown the reformatted oblique sagittal CT and 3DCT images. They could see slices of oblique sagittal CT images about all foramen as well as the foramen view by rotating to the left and right in 3DCT. They then noted any changes in the surgical plan based on the additional images. Each reviewer went through the same images and visit notes, which had been compiled into Microsoft Powerpoint presentation slides (Fig. 4). Reviewers were blinded to all patient identifiers, operative procedures and inclusion criteria.

### Statistical procedures

We analyzed the foraminal levels that were decompressed using anterior cervical discectomy and fusion, artificial disc replacement, posterior lateral fusion, or laminoplasty, the mean percent change in the surgical plan and the inter-observer variation in the decompressed foramen site. Kappa–Fleiss coefficients were calculated to compare reviewers’ surgical decisions. We evaluated the answer data pertaining to which foramen was decompressed using the Landis and Koch guidelines^20^ for interpreting kappa values, with values 0.0–0.2 indicating slight agreement, 0.21–0.40 indicating fair agreement, 0.41–0.60 indicating moderate agreement, 0.61–0.80 indicating substantial agreement and 0.81–1.0 indicating almost perfect or perfect agreement. R 2.8.1 (The R Foundation for Statistical Computing, Vienna, Austria) was used for statistical analysis of surgery plans.

### Approval

This study was approved by the Institutional Review Board of Columbia University, New York, U.S.A (AAAQ9561).

### Accordance

All methods were in accordance with the relevant guidelines and regulations.

### Informed consent

Informed consent was obtained from all participants.

## Data Availability

The datasets generated and/or analyzed during the current study are available from the corresponding author on reasonable request.
